# Target sequencing reveals genetic diversity, population structure, core-SNP markers, and fruit shape-associated loci in pepper varieties

**DOI:** 10.1186/s12870-019-2122-2

**Published:** 2019-12-23

**Authors:** Heshan Du, Jingjing Yang, Bin Chen, Xiaofen Zhang, Jian Zhang, Kun Yang, Sansheng Geng, Changlong Wen

**Affiliations:** 1 Beijing Vegetable Research Center (BVRC), Beijing Academy of Agricultural and Forestry Sciences, Beijing, 100097 China; 2 Beijing Key Laboratory of Vegetable Germplasm Improvement, National Engineering Research Center for Vegetables, Beijing, 100097 China; 3 Institute of Vegetables and Flowers, Chinese Academy of Agricultural Sciences, Beijing, 100081 China

**Keywords:** Pepper, SNP, Genetic structure, Target SNP-seq, Association analysis

## Abstract

**Background:**

The widely cultivated pepper (*Capsicum* spp.) is one of the most diverse vegetables; however, little research has focused on characterizing the genetic diversity and relatedness of commercial varieties grown in China. In this study, a panel of 92 perfect single-nucleotide polymorphisms (SNPs) was identified using re-sequencing data from 35 different *C. annuum* lines. Based on this panel, a Target SNP-seq genotyping method was designed, which combined multiplex amplification of perfect SNPs with Illumina sequencing, to detect polymorphisms across 271 commercial pepper varieties.

**Results:**

The perfect SNPs panel had a high discriminating capacity due to the average value of polymorphism information content, observed heterozygosity, expected heterozygosity, and minor allele frequency, which were 0.31, 0.28, 0.4, and 0.31, respectively. Notably, the studied pepper varieties were morphologically categorized based on fruit shape as blocky-, long horn-, short horn-, and linear-fruited. The long horn-fruited population exhibited the most genetic diversity followed by the short horn-, linear-, and blocky-fruited populations. A set of 35 core SNPs were then used as kompetitive allele-specific PCR (KASPar) markers, another robust genotyping technique for variety identification. Analysis of genetic relatedness using principal component analysis and phylogenetic tree construction indicated that the four fruit shape populations clustered separately with limited overlaps. Based on STRUCTURE clustering, it was possible to divide the varieties into five subpopulations, which correlated with fruit shape. Further, the subpopulations were statistically different according to a randomization test and *F*_*st*_ statistics. Nine loci, located on chromosomes 1, 2, 3, 4, 6, and 12, were identified to be significantly associated with the fruit shape index (*p* < 0.0001).

**Conclusions:**

Target SNP-seq developed in this study appears as an efficient power tool to detect the genetic diversity, population relatedness and molecular breeding in pepper. Moreover, this study demonstrates that the genetic structure of Chinese pepper varieties is significantly influenced by breeding programs focused on fruit shape.

## Background

Pepper are members of the genus *Capsicum*, which originated in South America and represents one of the most economically important vegetable crops worldwide [1–3]. To date, 38 species of *Capsicum* have been reported (USDA-ARS, 2011). Of these, *C. annuum*, *C. frutescens*, *C. chinense*, *C. baccatum*, and *C. pubescens* are thought to have been domesticated [4]. Globally, the most predominant species is *C. annuum*, which has numerous commercial varieties varying greatly in size, shape, pungency, and color.

As the seed trade has developed and globalized, the commercial quality of seeds, which is based on authenticity and purity, has become increasingly important [5]. Traditionally, cultivar characterization was completed by field investigation of morphological traits; however, this process is time-consuming and labor-intensive and is thus not suitable for modern inspection demands [6]. A more high-throughput approach to distinguish varieties is the used of molecular markers [5]. Indeed, genetic markers have been used for DNA fingerprinting, diversity analysis, variety identification, and marker-assisted breeding of multiple commercial crops [7, 8]. Moreover, several PCR-based tools have been used to detect genetic diversity in peppers, including random amplified polymorphic (RAPD), restriction fragment length polymorphism (RFLP), and amplified fragment length polymorphism (AFLP) [9–12].

Recently, the genomes of two *C. annuum* cultivars, Zunla-1 and CM334, were sequenced [3, 13], which provided an important platform for the detection and development of genome-wide simple sequence repeats (SSR) and insertion or deletion (InDel) markers [14–20]. Although a large number of SSR and InDel markers have become available, these technologies are not suitable for large scale germplasm characterization. Thus, there is an unmet need for an efficient, rapid, and high-throughput system capable of characterizing thousands of germplasm.

One approach for meeting such high standards is the use of single-nucleotide polymorphisms (SNPs), which are good markers for genotyping because of their whole genome coverage and primarily biallelic nature. Accordingly, multiple high-throughput SNP genotyping platforms have been developed, including the GoldenGate [21] and Infinium [22], TaqMan [23], and KASPar platform (KBiosciences, www.kbioscience.co.uk). In recent years, high-throughput transcriptome sequencing and genotyping-by-sequencing (GBS) have been successfully used in pepper, generating highly informative genome-wide SNP data [24–30]. However, SNP marker genotyping is considered expensive as it requires a comprehensive technical platform and special equipment and reagents.

Genotyping by target sequencing (GBTS) is a targeted sequence-capture strategy that can genotype more than thousands of SSRs or SNPs using high-throughput sequencing technology. The two main types of GBTS are multiplex PCR and probe-in-solution-based target sequencing; the technology has been commercialized as AmpliSeq [31], NimbleGen [32], SureSelect [33], GenoBaits, and GenoPlexs [34]. To date, this technology has been widely used for medical applications but has rarely been used for agriculture species. However, a Target SSR-seq technique, which is a multiplex PCR-based approach, was successfully applied to the study of genetic diversity and structure in 382 cucumber varieties [35]. The results of this study demonstrated that GBTS is a customizable, flexible, high-throughput, low cost, and accurate sequencing tool.

Peppers from China constitute one-third of the world’s pepper production [36]. Until now, the genetic diversity of pepper accessions in China has primarily been investigated using SSR markers, but these surveys only examined either several Chinese germplasm (up to 32) [37] or a small number of SSR markers (up to 28) [36]. However, high-throughput SNP platforms used for genotyping and the identification of pepper varieties have lagged significantly behind those for SSRs, and studies on the genetic diversity between the varieties of peppers in China has not yet been extensively analyzed. Therefore, the main objectives of the present work were: 1) to develop a Target SNP-seq technique suitable for genotyping pepper varieties; 2) to characterize composite core-SNP markers for use with the KASPar platform to maximize variety identification; 3) to examine the level of genetic diversity, structure, and differentiation within 271 pepper varieties. This study demonstrated that a novel Target SNP-seq can be used as a rapid and efficient tool for genotyping peppers, and the genetic structure of these cultivated varieties have been strongly impacted by breeding programs that select for fruit shapes.

## Results

### Genome-wide perfect SNPs used for target SNP-seq

**Fig. 1:**
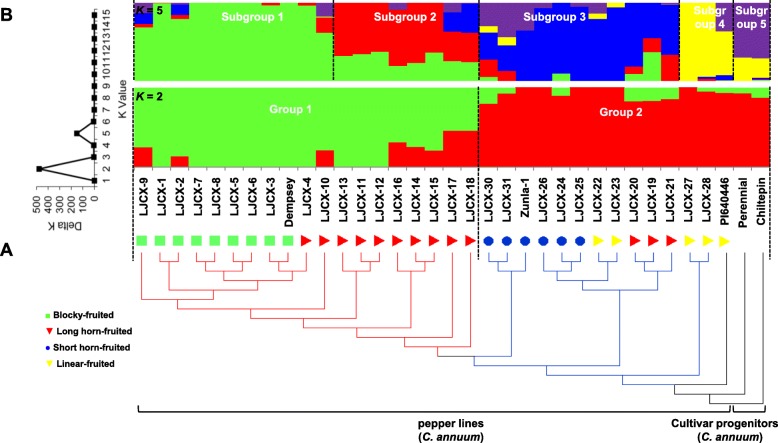
Population structure across pepper lines. Phylogenetic relationships (**a**) and population structure (**b**) based on the total SNPs of the 31 pepper inbred lines sequenced in this study and the previously sequenced *C. annuum* cultivars Zunla-1, Chiltepin [3], Perennial, and Dempsey [13]. Fruit shapes are presented as colored shapes

Re-sequencing of the 31 pepper lines (*C. annuum*) in this study generated a total of 872 Gb of paired-end sequence data, at an average depth of ~ 8.4. After mapping to the Zunla-1 genome [3], 40,700,040 SNPs were detected across the genomic sequences of the 31 re-sequenced lines and four previously published cultivars (Dempsey, Zunla-1, Perennial, and Chiltepin) [3, 13]. Approximately 11.3% of the *C. annuum* genome contains variable SNP sites. A total of 21,237,194 SNPs, with minor allele frequency (MAF) > 5% and missing data < 10%, were considered high-quality SNPs for downstream analyses. Using *C. annuum*’s progenitor cultivar, Chiltepin, as an outgroup, the phylogenetic tree showed that pepper lines could generally be classified according to fruit shapes, except for three long horn-fruited lines that grouped with the linear-fruited lines. Based on the genetic distance, the transitions in fruit shapes were from Chiltepin-like peppers followed by the linear-fruited, short horn-fruited, long horn-fruited, finally to blocky-fruited peppers, which were the furthest from the Chiltepin-like peppers (Fig. 1a). Furthermore, the 35 lines can be divided into two major groups based on the optimal number of *K* = 2 by STRUCTURE (Fig. 1b); Group 1 consisted of the nine bell-fruited lines and ten of the long horn-fruited lines, whereas the remaining peppers, including three long horn-fruited, all the linear-fruited, and all the short horn-fruited peppers, as well as two cultivar progenitors Perennial and Chiltepin were assigned to Group 2. The clustering of these pepper lines appeared to be more related to fruit type, when *K* = 5. Group 1 was divided into Subgroup 1 (mostly blocky-fruited) and Subgroup 2 (long horn-fruited), whereas Group 2 was composed of Subgroup 3 (admixture, mostly short horn-fruited), Subgroup 4 (linear-fruited), and Subgroup 5 (cultivar progenitors with small fruit).

**Fig. 2:**
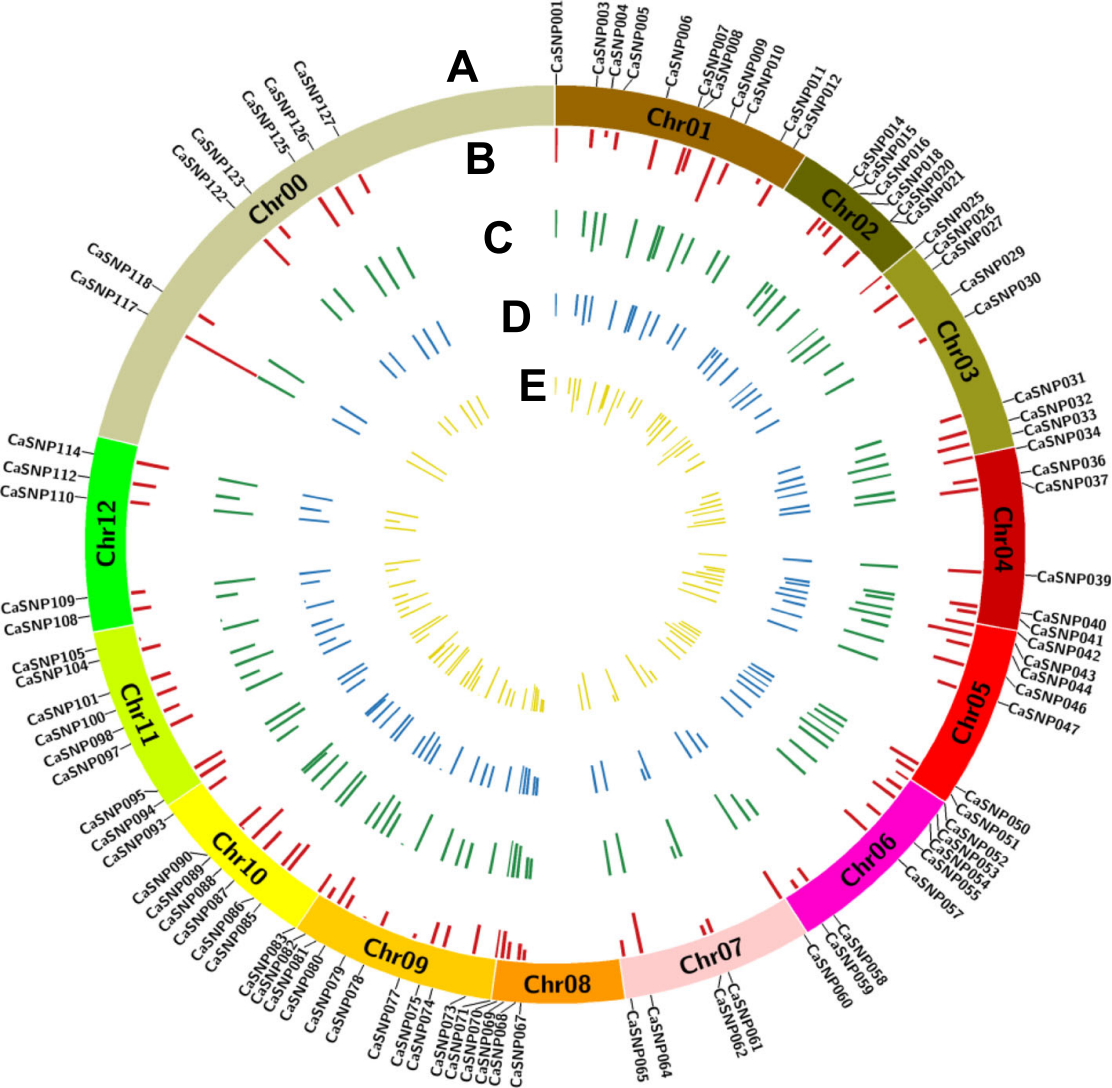
Characteristics of the perfect SNPs used to genotype pepper varieties by Target SNP-seq. **a** Distribution of the 92 perfect SNPs in the ideogram of the genome of *C. annuum* Zunla-1 [3]. **b** Observed heterozygosity (*Ho*) per SNP locus, colored in red. **c** Expected heterozygosity (*He*) per SNP locus is presented in green. **d** Polymorphism information content (PIC) per SNP locus is presented in blue. **e** Minor allele frequency (MAF) per SNP locus is given in yellow. This figure was generated using Circos (http://circos.ca/) with the SNP region magnified to 2 Mb

Given that pepper genomes are highly repetitive, strict criteria were used to identify the perfect SNPs (See Methods). In total, 521 perfect SNPs were identified, and 92, which were distributed across the genome (Fig. 2; Additional file 9: Table S2), were selected as multiplex PCR targets. Based on the previous annotation [3], 83 and 9 perfect SNPs fall within intergenic and genic regions, respectively. The nearest flanking annotated genes for each perfect SNP are shown in Additional file 9: Table S2.

### Genotyping analysis of pepper varieties using the target SNP-seq

In total, 271 pepper varieties, including 90 blocky-, 113 long horn-, 25 short horn-, and 43 linear-fruited varieties, were genotyped using the Target SNP-seq (Additional file 8: Table S1). A total of 55.9 million reads were generated from the 271 varieties, with an average target read depth of 2064, and approximately 82% of the samples were sequenced at a depth greater than 1000 × (Additional file 2: Figure S2A). Among the 271 varieties, 238 varieties (87.8%) aligned to the Zunla-1 genome [3] at a rate of more than 90% (Additional file 2: Figure S2B). Of these aligned reads, 221 varieties (81.5%) exhibited an align rate to the target SNP region of over 80% (Additional file 2: Figure S2C). Furthermore, the Target SNP-seq uniformity index was analyzed, which was used to calculate the proportion of the coverage above 10% of the mean depth value for each variety. The average uniformity index in this study was 93.68% (Additional file 2: Figure S2D), which indicated a high uniformity of sequence depth among the 92 SNPs.

### Perfect SNPs in 271 pepper varieties

The genetic parameters, MAF, *Ho*, *He*, and PIC revealed by each perfect SNP are given in Additional file 10: Table S3. MAF is a measure of the discriminating ability of the markers; as such, the closer the MAF is to 0.5 for biallelic markers, the better discriminatory properties. In this study, 28.26% of perfect SNPs showed a MAF between 0.4 and 0.5, whereas only four SNPs had MAF below 0.1 (Additional file 3: Figure S3A). The *Ho* value of each SNP ranged from 0.01 (CaSNP079) to 0.59 (CaSNP009) with an average of 0.28, and 11 SNPs exhibited higher *Ho* (> 0.4) (Additional file 3: Figure S3B; Additional file 10: Table S3). Furthermore, the *He* values ranged from 0.01 (CaSNP079) to 0.5 (CaSNP043 and CaSNP094) (Additional file 3: Figure S3C; Additional file 10: Table S3), whereas PIC values varied among perfect SNPs from 0.01 (CaSNP079) to 0.38 (CaSNP043, CaSNP094 and CaSNP117) with a mean of 0.31 (Additional file 3: Figure S3D; Additional file 10: Table S3). 71.74% of the perfect SNPs had PIC values greater than 0.30, whereas only four SNPs showed PIC values below 0.2. These values indicate that the perfect SNPs panel has a high discriminating capacity for varieties, and that CaSNP043, CaSNP94, CaSNP117, and CaSNP009 were the best at discriminating between varieties. Overall, the results indicate that the Target SNP-seq can be used as a rapid tool for genotyping peppers.

### Perfect SNPs across the fruit shapes

The average values of the genetic parameters across the four fruit shape populations were also compared for genetic diversity, and the results showed that the blocky-fruited population had the lowest average values for *He* (0.18), *Ho* (0.16), and PIC (0.15) (Table 1), indicating the lowest genetic diversity within this population. In contrast, the long horn-fruited population exhibited the highest genetic diversity as defined by the highest average values of *He* (0.39), *Ho* (0.36), and PIC (0.31).

**Table 1:** Genetic diversity in fruit shape populations and across all varieties

Fruit shapes	Varieties size	PIC^a^	*He*	*Ho*	MAF
Blocky-fruited	90	0.15 (4)^b^	0.18 (4)	0.16 (4)	0.13 (4)
Long horn-fruited	113	0.31 (1)	0.39 (1)	0.36 (1)	0.30 (1)
Short horn-fruited	25	0.29 (2)	0.37 (2)	0.34 (2)	0.29 (2)
Linear-fruited	43	0.25 (3)	0.31 (3)	0.33 (3)	0.23 (3)
Total	271	0.31	0.40	0.28	0.31

^a^For each fruit population: polymorphism information content (PIC), expected heterozygosity (*He*), observed heterozygosity (*Ho*), and minor allele frequency (MAF)

^b^The numbers in parentheses refer to the numerical ranking of diversity in a descending order

A total of 21 SNP loci did not indicate any diversity (PIC = 0) within certain fruit populations, of which 16, 1, 3, and 5 loci were for the blocky-, long horn-, short horn-, and linear-fruited population, respectively (Additional file 10: Table S3). These fruit shape-specific loci may have been under selection during breeding or were selected owing to linkage with genes that determine fruit traits.

### Identification of a core-SNP set

**Fig. 3:**
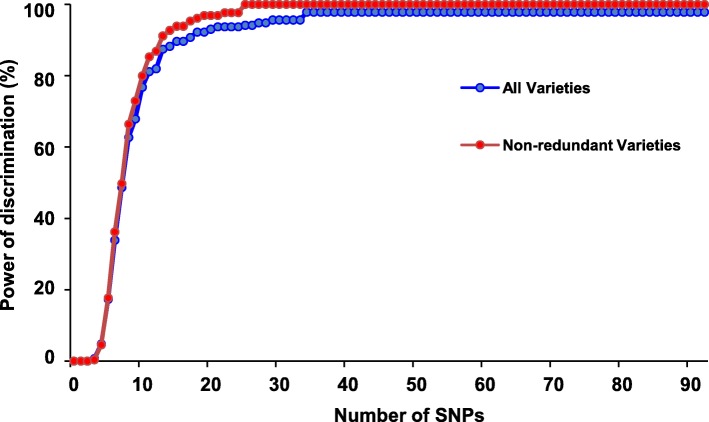
Discriminating saturation curve of 92 perfect SNPs in pepper varieties. The maximum discrimination power was 97.7% across all 271 varieties using 35 perfect SNPs, and 100% across non-redundant varieties using 27 perfect SNPs

The perfect SNP panel distinguished 97.7% of the 271 pepper varieties (Fig. 3), the remaining displayed the same multilocus genotypes that were also difficult to distinguish from field phenotypes. Given that some varieties may exist with multiple names, varieties with identical genotypes may be redundant and were discarded to build non-redundant genotype varieties. Thus, a minimum of 27 perfect SNPs could distinguish between all non-redundant varieties (Fig. 3).

To develop a core-SNP set for the KASPar platform, each perfect SNP marker was tested on a set of 23 to 95 pepper varieties with two allele-specific forward primers and one common reverse primer. The results showed that 35 SNP primers (Additional file 11: Table S4; Additional file 4: Figure S4) produced consistent and repeatable results with Target SNP-seq. Finally, 35 SNPs with a high discrimination power of up to 97% across all varieties and 100% in non-redundant varieties were proposed as a core SNPs set for use with the KASPar platform (Fig. 3 and Additional file 4: Figure S4; Additional file 11: Table S4).

### Genetic structure in pepper varieties

**Fig. 4:**
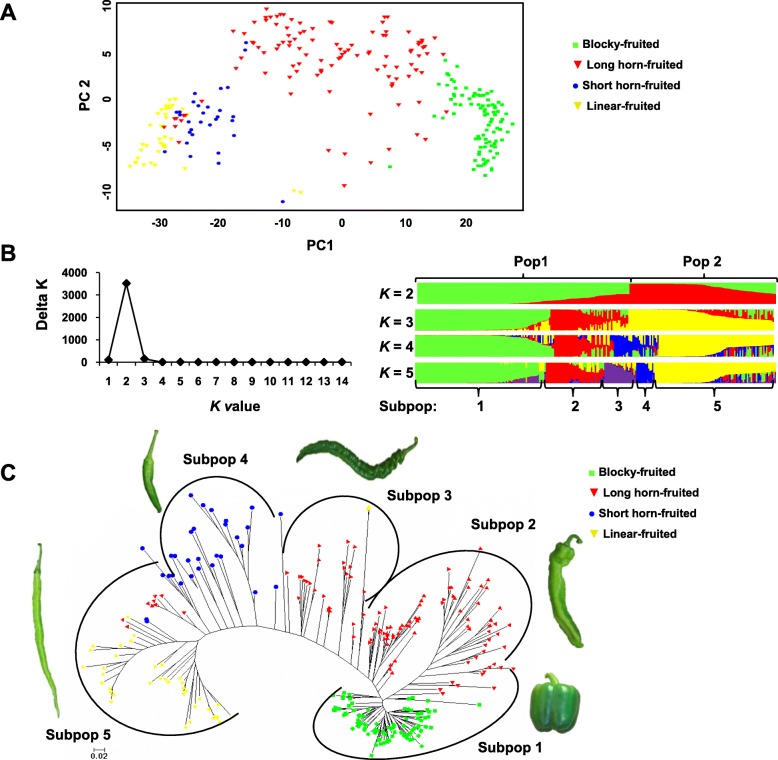
Population structure across pepper varieties. **a** Principal component analysis (PCA). **b** Population structure inferred using STRUCTURE. All varieties were divided into two main populations (Pop1 and Pop2) when *K* = 2, which was the optimal *K*. The populations were subdivided into five subpopulations, Subpop1~Subpop5, which correlated with fruit shape. **c** Phylogenetic tree analysis. The tree was produced using the neighbor-joining method based on the 92 perfect SNPs. The scale bar indicates simple matching distance

The principal component analysis (PCA) was performed using the 92 perfect SNPs to investigate population clusters across the 271 varieties (Fig. 4a). Accordingly, the PCA plot indicates that the four fruit shape populations were generally clustered separately. The distribution of blocky-fruited varieties was very concentrated, whereas that of the long horn-fruited varieties was relatively dispersed. Linear-fruited varieties were more closely related to the short horn-fruited varieties than to either the long horn- or blocky-fruited varieties. Linear and blocky-fruited populations were the most diverse, and these clusters did not overlap, suggesting considerable genetic divergence throughout their breeding history. Notably, a selection of both long horn- and short-fruited varieties showed close relatedness to the linear-fruited population.

The population structure of the 271 varieties was further inferred using the cluster program, STRUCTURE, testing for 2 to 5 number of clusters (*K*). Evanno’s correction [38] showed the peak of delta *K* at *K* = 2, which suggests the presence of two main populations, denoted as Pop1 and Pop2. Pop1 comprised 160 varieties (59.0%), containing all blocky-fruited varieties, 60.2% of long horn-fruited varieties, and only two linear-fruited varieties (Fig. 4b; Additional file 8: Table S1). The remaining 111 varieties (41.0%) were assigned to Pop2, which included all the short horn- and linear-fruited varieties, as well as 39.8% of long horn-fruited varieties (Fig. 4b; Additional file 8: Table S1). When *K* = 3, Pop1 was subdivided into two clusters, blocky- or long horn-fruited types. At *K* = 4, a mixture of 56% short horn-, 15 long horn-, and two linear-fruited varieties were assigned to a new cluster from Pop2, and these short horn- and a new long horn-fruited groups were assigned to independent clusters, respectively, when *K* = 5. Of note, the linear-fruited types were never assigned to an independent cluster as *K* was increased. Considering that the classification of populations appeared highly correlated with fruit types when *K* = 5, the two main populations were further subdivided into five subpopulations (Subpop1~Subpop5; Fig. 4b; Additional file 8: Table S1). Subpop1, 2, 3, and 4 showed a clear-cut structure with no or very few admixtures. Subpop1 comprised 98 varieties, 90 of which belong to blocky-fruited varieties and the remaining eight to long horn-fruited varieties. Long horn-fruited varieties were members of both Subpop2 and Subpop3, which is not surprising as long horn-fruited varieties were distributed across both Pop1 and Pop2. Subpop2 comprised 44 long horn-fruited varieties. Subpop3 comprised 24 varieties, 22 of which were long horn-fruited varieties and the remaining two were linear-fruited varieties. Subpop4 comprised 14 short horn-fruited varieties. Consistent with the results of PCA analyses, admixtures were mostly located in Subpop5, which contained 41 long linear-fruited varieties as well as a minority of short horn- and long horn-fruited varieties.

The unrooted phylogenetic tree (Fig. 4c) is consistent with the aforementioned PCA and model-based population structure, and indicated a clear distinction in the four fruit shapes, despite having admixtures. Images of the representative varieties, which were selected based on the lowest average genetic distance to other varieties within corresponding subpopulations, are presented in Fig. 4c. The representative images for two long horn-fruited varieties from Subpop2 and Subpop3 clearly indicate distinct morphologies.

In summary, three independent analysis methods strongly supported the division of pepper varieties into five well-differentiated genetic populations, which were correlated with distinct fruit shapes, indicating that the genetic structure of these cultivated varieties may have been strongly affected by fruit shape selection through breeding practices.

### Genetic variation assessment of pepper populations

Comparison of the results between Pop1 and Pop2 using analysis of molecular variance (AMOVA) revealed that 33.04% of the total genetic variation was partitioned among Pops, 8.47% within Pops, and the remaining 58.49% within varieties (Table 2). AMOVA analysis of the five Subpops further indicated that the maximum variation (63.83%) occurred within varieties, the minimum variation (3.54%) was accounted for within Subpops, and 32.63% of the variation occurred between Subpops (Table 2), suggesting relatively moderate differentiation among Subpops.

**Table 2:** Analysis of molecular variance (AMOVA) among Pops and Subpops

	Sum of squares	Variance components	Percentage of variation
Among Pops / Subpops	4013.64 / 5368.25	14.88 / 13.46	33.04% / 32.63%
Within Pops / Subpops	9137.06 / 7782.44	3.81 / 1.46	8.47% / 3.54%
Within varieties of Pops / Subpops	7137.53 / 7137.53	26.34 / 26.34	58.49% / 63.83%
Total	20,288.23 / 20,288.23	45.03 / 41.26	100.00 / 100.00%

To test for significant variations between Pops and among Subpops, a randomization test was performed (Additional file 5: Figure S5). The output revealed six histograms representing the distribution of the randomization strata. The observed results in the output showed significant differentiation of the structure of Pops and Subpops considering all levels of Pops and Subpops strata (Additional file 5: Figure S5). These results also supported the separation of the varieties into two Pops and five Subpops. Furthermore, pairwise estimates of *F*_*st*_ showed that population differentiation between Pop1 and Pop2 was high (*F*_*st*_ = 0.35). The pairwise *F*_*st*_ between the five Subpops ranged from 0.13 between Subpop2 and Subpop3 (both consist largely of long horn-fruited varieties) to 0.48 between Subpop1 (mostly blocky-fruited varieties) and Subpop4 (short horn-fruited varieties) (Table 3). Notably, high genetic differentiation (*F*_*st*_ = 0.43) was observed between Subpop1 and Subpop5 (mostly consisting of linear-fruited varieties), whereas lower genetic differentiation was observed between Subpop4 and Subpop5 (*F*_*st*_ = 0.14).

**Table 3:** Pairwise *F* statistics (*F*_*st*_) estimates among subpopulations

Subpopulations	Subpop2	Subpop3	Subpop4	Subpop5
Subpop1	0.21	0.23	0.48	0.43
Subpop2		0.13	0.31	0.28
Subpop3			0.22	0.18
Subpop4				0.14

### Identification of the loci associated with fruit shape

A wide range of variation was observed for fruit shape index (FSI) in the 271 pepper varieties (Additional file 12: Table S5). The average FSI was 1.34, 4.98, 4.70, and 16.56 in blocky-, long horn-, short horn-, and linear-fruited populations, respectively. Significant differences were observed among blocky-, horn-, and linear-shaped populations (*p* < 0.01), but no differences were detected between long horn and short horn-fruited populations. FSI values of more than 9.5 are typical of linear fruits.

Having observed concordance between the population structure and fruit shapes (Fig. 2), we next performed association analyses of the FSI in 271 varieties and 165 genetic loci, including 92 SNPs and an additional 73 SSRs, which were all detected using Target sequencing (Additional file 6: Figure S6). Using the *K + Q* mixed linear model (MLM), a total of nine loci (CaSSR013, CaSSR090, CaSSR105, CaSSR091, CaSSR039, CaSSR044, CaSSR107, CaSSR077, and CaSNP112) were identified as significantly associated with FSI under a threshold *p*-value of 0.0001 (Additional file 7: Figure S7; Additional file 13: Table S6). To pair the associations with previously identified quantitative trait loci (QTL), the physical position of the nine loci in both the reference genome of Zunla-1 [3] and CM334 [13] are provided in Table 4. Loci CaSSR091 and CaSSR039 are within 820 kb on the same chromosome and were considered a unit. Therefore, the nine loci were located at eight chromosomal regions on six chromosomes, including chromosomes 1, 2, 3, 4, 6, and 12, and the phenotypic variation explained by each locus ranged from 7.9 to 12.7%. Two loci, CaSSR044 and CaSSR107, spanning approximately 39 Mb on chromosome 6, explained the highest phenotypic variation, which was 12.4 and 12.7%, respectively (Table 4 and Additional file 13: Table S6; Additional file 7: Figure S7).

**Table 4:** Loci significantly associated with fruit shape index as identified by association analysis

Loci^a^	CM334 (v.1.55) [13]		Zunla-1 (v2.0) [3]		*p*-value	Phenotypic variation explained (%)
	Chr.	Physical position (bp)	Chr.	Physical position (bp)		
CaSSR013	1	2,895,632	1	2,383,961	2.16E-06	9.7
CaSSR090	2	153,032,594	2	143,851,440	1.36E-06	8.2
CaSSR105	3	143,624,183	3	95,195,822	6.99E-05	9.1
CaSSR091	4	762,569	4	214,859,753	2.17E-05	8.9
CaSSR039	4	ND	4	215,677,699	1.99E-05	8.9
CaSSR044	6	234,686,966	6	1,041,327	6.95E-08	12.7
CaSSR107	6	193,777,105	6	39,731,428	1.88E-05	12.4
CaSSR077	12	226,618,758	12	9,517,953	7.54E-08	10.1
CaSNP112	12	47,349,405	12	181,587,490	8.45E-06	7.9

^a^The interval between CaSSR091 and CaSSR039 was within ~ 820 kb; thus, these two loci were considered at the same associated region

## Discussion

### High-throughput genotyping by target SNP-seq

High-throughput genotyping technology has become essential for effective crop breeding programs. Target SSR-seq, which combined the multiplexed amplification of perfect SSRs with high-throughput sequencing, was recently developed and applied to the identification of cucumber varieties, leading to the characterization of a set of core SSRs [35]. This sequencing technology can acquire thousands of data points in under 72 h, costs less than $7/sample, and is associated with genotyping accuracy up to 100% due to the high coverage. The cost of Target SNP-seq developed in this study was similar as that of Target SSR-seq because the same procedure was used for target library construction in these two technologies.

In this study, re-sequencing tools were used to identify 92 perfect SNPs from the genomes of 35 *C. annuum* based on strict screening criteria*.* Only 9.8% of the perfect SNPs fell within genic regions, which is in agreement with the previous result that variant density is significantly lower in the genic region than in the intergenic regions [30]. The identified perfect SNPs were then used for target SNP sequencing to assess genetic diversity across 271 pepper varieties that are popular in China. The results showed that the perfect SNP panel had a high discriminating capacity for varieties, as 71.74% of the perfect SNPs had PIC values of > 0.30 (Additional file 10: Table S3). Further, a minimum of 27 perfect SNPs could distinguish between all non-redundant varieties (Fig. 3). Notably, the mean PIC value was found to be 0.31, which is lower than the values derived from studies using SSR markers [17, 39]. These discrepancies may be explained by the nature of the different types of markers; SSRs are multiallelic and more polymorphic than SNP markers, which are biallelic [40]. Another reason for the discrepancies may be due to the commercial varieties tending to be less variable compared to the landraces or the wide germplasm collection.

A set of 35 core SNPs that had the same discrimination power as the 92 perfect SNPs was successfully converted into KASPar markers, representing another robust genotyping choice for pepper varieties (Fig. 3; Additional file 11: Table S4). Unlike SSR markers, SNP markers do not require reference cultivars to be included in each experiment and will also overcome the confusion between labs regarding SSR alleles.

### Population structure among inbred *C. annuum* lines

Since their initial domestication in Mexico, peppers have been under strong selection for fruit shape and size [56]. Consumption habits and pepper type preference vary globally. In the US alone, more than 20 market types are recognized and consumed [57]. In China, most of the pepper varieties commercially cultivated belong to the species *C. annuum*, and the market types are classified by fruit shapes, such as the popular blocky, long horn, short horn, and linear fruits [58, 59]. To date, most experiments have evaluated the genetic relationships among several *Capsicum* species [29, 30, 36, 37, 60–62] or the genetic diversity of *C. chinense* and *C. baccatum* germplasm from relatively restricted regions [26, 63]. Phylogenetic analysis based on molecular markers, pan-genome sequencing, and GBS confirmed that *C. chinense* and *C. frutescens* are more closely related to each other than to *C. annuum* [29, 61, 64]. Several studies attempted to characterize the population relatedness of cultivated *C. annuum* in restricted geographical areas [29, 36, 40–42, 65]. They revealed that the population structures of the *C. annuum* accessions were mainly associated to distinct cultivar types with respect to the plant and fruit descriptors, and thus mostly result from human selection for cultivar types in agreement with consumption modes and adaptation to the highly diversified agro-climatic conditions. Notably, the relationships among the 35 re-sequenced *C. annuum* lines described in this study align with previous reports grouping *C. annuum* according to fruit traits [29, 41, 65]. Further, clustering of blocky-fruited peppers in the furthest positions relative to small hot Chiltepin-like types can also be observed in previous studies [29, 41, 65].

### Genetic structure among *C. annuum* varieties

Although the previous work has shown that population of *C. annuum* landraces in China clusters according to cultivar type [36], the relationships among commercially important *C. annuum* varieties from different companies have not been investigated with a fine set of genetic markers. In the present study, the relationships among four fruit shape populations were assessed across a broad range of pepper varieties cultivated in China. Comparison of the genetic parameters showed the lowest *Ho* was observed within the blocky-fruited population, while the highest was detected in the horn-fruited population (Table 1). These findings agree with the earlier studies that found a reduction in diversity was associated with non-pungent blocky-fruited lines relative to pungent lines [41–44]. The narrow genetic diversity associated with the blocky-fruited varieties may be a consequence of inbreeding with a limited gene pool.

Additionally, the PCA and phylogenetic tree demonstrated that the four fruit shape populations clustered separately with a little or no overlap. This aligns with the fruit shape classification system and demonstrates that the genetic structure of pepper varieties in China has been significantly influenced by breeding programs that select for fruit shape. Similarly, STRUCTURE analysis grouped the varieties into two main populations, Pop1 and Pop2, which were further divided into five subpopulations, Subpop1 to Subpop5 (Fig. 4b). Moreover, the subpopulations correlated with fruit shape. Notably, Subpop1, Subpop4, and Subpop5 corresponded to the blocky, short horn, and linear-fruited varieties, respectively. However, the majority of the long horn-fruited varieties were divided into two subpopulations, Subpop2 and Subpop3, which were statistically unique (Additional file 5: Figure S5). The best fit of genetic structures of the pepper lines and varieties were both divided into two groups in this study, different to that observed in the 368 Chinese *C. annuum* accessions analyzed by Zhang et al. [36, 18], which included 28 SSR markers to structure the accessions into three STRUCTURE groups. These differences may be attributed to the different types of pepper materials and the number of markers used in the two studies. However, the clustering of fruit types in both studies appeared to be somewhat similar, although different classifications of fruit shape were used. For example, Group1 mainly included rectangular, square, and triangular fruit types [36], which were also mainly clustered in Pop1 of our study. Group 3 mainly comprised cultivars with small and long fruits characterized by a very high fruit length: width ratio [36], which is the characteristic of linear-fruited and some short horn-fruited varieties in Pop2 of this study. In summary, our study provided valuable insight into the population structure underlying the fruit shapes of pepper varieties, as well as confirmed the strong effect of fruit shape selection by breeders on the genetic structure of Chinese pepper varieties.

### Identification of associated loci for fruit shape

Fruit shape is an important trait in pepper breeding programs. A number of QTLs controlling FSI have been identified in intraspecific and interspecific populations from a cross between the bell pepper and a small-fruited hot pepper [45–53]. The first FSI QTL in peppers, named *fs3.1*, was detected on linkage group 3 [48]. This QTL was subsequently detected in other linkage analyses [46, 47, 51]. Using genome-wide associations in 373 pepper accessions, Colonna et al. (2019) recently identified that the SNP 3:183386147 on chromosome 3, located in the exon of gene CA03g16080, is significantly associated with FSI [30]. In the current study, the FSI associated loci CaSSR105 and its nearest upstream marker, CaSNP029, are located within approximately 26 Mb intervals on chromosome 3 (Additional file 13: Table S6). This FSI association region covers the reported FSI associated loci SNP 3:183386147 in the gene *Longifolia 1-like* (CA03g16080) [30]. Zygier et al. (2005) mapped a fruit shape QTL (*fs2.1*) on chromosome 2 [50]. This QTL was also detected in other studies and was found to be close to the *Ovate* gene (CA02g22830) at approximately 158 Mb of the CM334 genome [52, 53]. In the current study, FSI associated loci CaSSR090 and its nearest downstream loci CaSSR024, with an interval spanning of approximately 12.6 Mb on chromosome 2, covered the reported *Ovate* gene (CA02g22830). The *Ovate* gene was initially discovered in the tomato, where it controlled the fruit shape transformation from round to pear-shaped fruit [54, 55]. A single FSI QTL (*fs4.2*) was detected at the end of chromosome 4, which explained the 26.1% phenotypic variation [50]. We found that two FSI associated loci, CaSSR091and CaSSR039, were located between CaSNP041 and the end of chromosome 4, covering approximately 12.9 Mb (Additional file 13: Table S6). The presence of FSI association loci on chromosomes 1, 6, and 12 was also detected in this study (Additional file 6: Figure S6 and Additional file 7: Figure S7; Table 4 and Additional file 13: Table S6). After screening for protein function in these association loci, we found that two *Ovate* genes, CA06g21580 and CA12g07370, could be considered candidate genes, as they had significant effects on fruit shape.

### Future directions of target SNP-seq in pepper

Of note, foreground selecting markers that are suitable for specific primer design could also be added to the perfect SNP panel used in our Target SNP-seq. For example, based on the functional site of the *Tobamovirus* resistance gene *L*^*3*^ and *L*^*4*^ [66], the *Phytophthora capsici* resistance genes *CaDMR1* and Phyto5NBS1 [67, 68], bacterial spot resistance gene *Bs3* [69], and *potato virus Y* resistance gene *pvr1* [70], specific primers at the flanking region of the functional site have successfully been developed (Additional file 14: Table S7) and added to the perfect SNP panel. These functional loci of resistance genes, combined with the perfect SNP and SSR markers, could be detected simultaneously across hundreds of pepper accessions through Target SNP-seq. The commercial application of this technique has the potential to increase the efficiency of marker-associated selection programs, as well as aiding in variety identification.

## Conclusions

The Target SNP-seq developed in this study is a high-throughput and reliable tool for the investigation of genetic diversity, variety identification, and characterization of population structure in peppers. The use of PCA, phylogenetic tree generation, and STRUCTURE revealed that the genetic structure of commercially available pepper varieties in China had been significantly influenced by fruit shape selection through breeding. Finally, association analysis of a limited number of markers allowed for the identification of previously reported and novel genomic regions that control fruit shape.

## Methods

### Plant materials, fruit shape categorization, and DNA extraction

A total of 271 pepper varieties, which were kindly supplied by 60 different breeding companies in China, were analyzed in this study. Information on these hybrid seeds, including variety name and source, is available in Additional file 8: Table S1. Fruit trait investigation and genetic identification were carried out by the pepper genetic breeding group and high-throughput molecular breeding platform at the Beijing Vegetable Research Center (BVRC).

Varieties were planted under greenhouse conditions at the Vegetable Varieties Exhibition Center in the Tongzhou District of Beijing. The greenhouse temperature ranged from 25 to 30 °C (08:00–20:00) and 20–25 °C (20:00–08:00), with natural light. Each variety consisted of at least four plants. The fruits were categorized into one of four fruit shapes: blocky-, long horn-, short horn-, and linear-fruited types (Additional file 8: Table S1).Examples of fruit shape classification are presented in Additional file 1: Figure S1. Four to ten ripe fruits from each plant were subjected to measurements of maximum height and width using a Vernier caliper (Hangzhou Tool and Measuring Tool Company, Hangzhou, China).

DNA was extracted from four young plantlet randomly selected from individuals of each variety using a CTAB-based method [71]. The DNA integrity was assessed using 1.5% (w/v) agarose gel electrophoresis, and the concentration was determined using a Nanodrop 2000 Spectrophotometer (Thermo Fisher Scientific, DE, USA).

### Re-sequencing and perfect SNP identification

In total, 31 diverse pepper lines (*C. annuum*), including 30 inbred lines from our ongoing breeding programs in BVRC and PI640446 provided by the U.S. National Plant Germplasm System, were selected for re-sequencing on the Illumina X Ten platform at Shanghai Majorbio Biopharm Technology Co. Ltd. (Shanghai, China). The 31 pepper lines had diverse genetic backgrounds and horticultural traits, including eight blocky-fruited lines, 13 long horn-fruited lines, five short horn-fruited lines and five linear-fruited lines (Fig. 1).

The raw reads of the 31 re-sequenced lines and four previously sequenced cultivars; Zunla-1 (*C. annuum*) and its wild progenitor Chiltepin (*C. annuum* var. *glabriusculum*) [3], *C. annuum* cv*.* Perennial and *C. annuum* cv*.* Dempsey [13], were filtered into clean data using Trimmomatic [72]. The clean reads were then mapped to the reference genome of Zunla-1 chromosome version 2.0 [3] using the Burrows-Wheeler Alignment Tool (BWA) with default parameters, and SNPs were called using the Genome Analysis Toolkit (GATK, v2.4-7g5e89f01) [73]. SNPs with MAF > 5% and missing data < 10% were imported into MEGA to build the rooted phylogenetic tree using the cultivar progenitor, Chiltepin, as an outgroup with the neighbor-joining method [74]. Population structure analysis was completed using STRUCTURE v2.3. The number of populations (*K*) was determined following the standard procedure [75] with a burn-in period of 100,000 iterations and Markov Chain Monte Carlo of 100,000. Twenty independent runs were performed for *K* varying from 1 to 15. The optimum *K* was defined according to Evanno’s delta *K* method [38].

To acquire a dataset of genome-wide SNPs for subsequent Target SNP-seq analysis, perfect SNPs were identified using the following criteria: (i) MAF > 0.4 to filter out uninformative SNPs; (ii) miss rate < 0.2; (iii) heterozygosity < 0.2; (iv) no sequence variation in the 100 bp flanking sequence of the SNP locus; and (v) 2 alleles per locus for the SNPs.

### Target SNP-seq

The Target SNP-seq procedure was completed as previously described using the SNPs identified above [35]. In brief, library construction for Target SNP-seq consisted of the following two rounds of PCR: the first round amplified and captured the target SNPs in DNA samples using the multiplexed panel of perfect SNP primers (Additional file 9: Table S2); the second round added a unique barcode to the capture product for each DNA sample. Thus, the samples are distinguished based on the different barcodes. The multiplexed PCR was conducted in a 30 μl reaction mixture, containing 50 ng genomic DNA template, 8 μl of the multiplexed SNP-capture panel primers (10 μM), 10 μl of 3 M enzymes (Molbreeding Biotechnology Company, Shijiazhuang, China). The PCR mixtures were heated at 95 °C for 5 min followed by 17 cycles at 95 °C for 30 s, 60 °C for 4 min, 72 °C for 4 min with a final 4 min extension at 72 °C. The PCR products were purified using a magnetic bead suspension and 80% alcohol. Similarly, the second PCR amplification was performed in a 30 μl reaction volume containing 11 μl of purified PCR product from the previous round, 10 μl of 3 M *Taq* enzyme (Molbreeding Biotechnology Company, Shijiazhuang, China), 8 μl nuclease-free water, and 1 μl of primers with the following sequences: forward 5′- AATGATACGGCGACCACCGAGATCTACACTCTTTCCCTACACGA -CGCTCTTCCG-3′ and reverse 5′-CAAGCAGAAGACGGCATACGAGAT -*XXXXXXXX*GTGACTGGAGTTCCTTGGCACCCGAGA-3′ (barcodes are indicated by underlined sequences). The PCR procedure was 95 °C for 3 min; 7 cycles of 95 °C for 15 s, 58 °C for 15 s, and 72 °C for 30 s with a final 4 min extension at 72 °C. The PCR products were then purified with 100 μl of 80% alcohol and 23 μl Tris-HCl buffer (10 mM, pH 8.0–8.5). After that, a Target SNP-seq library was sequenced using an Illumina X Ten platform at Molbreeding Biotechnology Company (Shijiazhuang, China).

### SNP genotype analysis of target SNP-seq

The raw data from the Target SNP-seq was de-multiplexed to determine the exact genotypes for each variety based on the sample-specific barcodes using the Illumina bcl2fastq pipeline (Illumina, San Diego, CA, USA). Clean data were filtered out using Trimmomatic, and the reads of each variety were mapped to the pepper reference genome of Zunla-1 [3] using BWA with default parameters. Sequence depth, alignment rate, as well as target alignment rate and uniformity for each variety were calculated as follows to evaluate the results of targeted sequencing.

$$ \mathrm{Alignment}\ \mathrm{rate}=\mathrm{the}\ \mathrm{number}\ \mathrm{of}\ \mathrm{reads}\ \mathrm{aligned}\ \mathrm{on}\ \mathrm{the}\ \mathrm{genome}/\mathrm{total}\ \mathrm{reads}. $$

$$ \mathrm{Target}\ \mathrm{alignment}\ \mathrm{rate}=\mathrm{the}\ \mathrm{number}\ \mathrm{of}\ \mathrm{reads}\ \mathrm{aligned}\ \mathrm{on}\ \mathrm{the}\ \mathrm{target}\ \mathrm{region}/\mathrm{total}\ \mathrm{reads}. $$

Uniformity inferred to the proportion of the SNPs with a depth of > 10% of the average depth.

$$ \mathrm{Depth}\ \mathrm{of}\ \mathrm{each}\ \mathrm{SNP}=\mathrm{total}\ \mathrm{base}\ \mathrm{generated}\ \mathrm{in}\ \mathrm{the}\ \mathrm{perfect}\ \mathrm{SNP}/\mathrm{read}\ \mathrm{length}. $$

$$ \mathrm{Average}\ \mathrm{depth}=\mathrm{S}/\left(\mathrm{M}\times \mathrm{N}\times \mathrm{L}\right), $$

where S indicates the total base generated from Target SNP-seq; M indicates the total number of varieties; N indicates the total number of SNPs; L indicates the average read length.

SNP genotypes were called using GATK. Based on the high-throughput sequencing results, the SNP alleles with the maximum numbers of reads and the second maximum numbers of reads were treated as the major and minor allele for each target SNP locus. When the read frequency of the major allele was higher than 0.7, the locus was described as homozygous. If the read frequencies of the major and minor allele were both more than 0.35, the locus was described as heterozygous.

Sequence depth for each variety = Total base generated from variety / total base length of the 92 targeted genome region.

### Determination of genetic parameters for each perfect SNP

Genetic parameter statistics of the perfect SNPs, including the observed heterozygosity (*H*_*o*_), expected heterozygosity (*H*_*e*_), and polymorphism information content (PIC) [76] were calculated using a Perl script with the following equation:

$$ \mathrm{PIC}=1-\sum \limits_{i=1}^l{P}_i^2-\sum \limits_{i=1}^{l-1}\sum \limits_{j=i+1}^l2{P}_i^2\ {P}_j^2 $$

where *l* is the allele locus, and *P*_*i*_ and *P*_*j*_ represent the population frequency of the *i*^*th*^ and *j*^*th*^ allele. The chromosomal distribution of the perfect SNPs was mapped using Circos software (http://circos.ca/) with the SNP region magnified to 2 Mb.

### Genetic structure analysis

Genetic relationships among varieties were investigated using three different methods: PCA, STRUCTURE, and a phylogenetic tree. PCA was carried out using the FactoMineR package in R [77]. The Bayesian-based model procedure implemented by STRUCTURE v2.3 [75, 78] was also used to determine population structure. The number of populations (*K*) was determined as described above, and the unrooted phylogenetic tree was constructed using the *Ape* and *Poppr* packages in R based on the neighbor-joining method with the tree viewed using MEGA v5.1 [79, 80].

### Population diversity analysis

The different fruit shape populations, as well as the subpopulations inferred from STRUCTURE, *Ho*, *He*, PIC, and MAF analyses, were calculated using the methods mentioned above. To measure genetic differences of populations and subpopulations, AMOVA and pairwise *F*_*st*_ were performed using *poppr* R package and the function pairwise.neifst in the *Hierfstat* R package, respectively [81, 82]. Randomization tests were further performed to test the significance of differentiation using the function randtest in the *ade4* package [81]. Detailed instructions for the AMOVA and randomization tests are available at https://grunwaldlab.github.io/Population_Genetics_in_R/AMOVA.html and https://rdrr.io/cran/poppr/man/poppr.amova.html.

### Discrimination power of the perfect SNPs

To determine a minimal number of SNPs for distinguishing the maximum number of pepper varieties, a Perl script was developed to determine the best discrimination power for 1 to 92 perfect SNPs according to the following algorithm.

1) Selection of the first SNP: a) pairwise comparison between varieties were conducted for each SNP, which included 36,585 comparisons for each SNP; b) X_ij_ = 1 if genotype difference existed for j^th^ pairwise comparison of i^th^ SNP (i = 1, 2, 3, …, 92; j = 1, 2, 3, …, 36,585). X_ij_ = 0 if genotype difference did not exist for j^th^ pairwise comparison of i^th^ SNP (i = 1, 2, 3, …, 92; j = 1, 2, 3, …, 36,585); c) The SNP with a maximum value of ${\sum}_{j=1}^{36585}\mathrm{Xij}$ among the 92 SNPs was selected as the first SNP. 2) Selection of the best two SNPs: a) 91 SNP combinations of the first selected SNP and each of the rest 91 SNPs were formed; b) 36,583 pairwise comparisons were conducted for each of the 91 SNP combination; c) X_mj_ = 1 if genotype difference existed for j^th^ pairwise comparison of m^th^ SNP combination (m = 1, 2, 3, …, 91; j = 1, 2, 3, …, 36,583). X_mj_ = 0 if genotype difference did not exist for j^th^ pairwise comparison of m^th^ SNP combination (m = 1, 2, 3, …, 91; j = 1, 2, 3, …, 36,583); d) the SNP combination with a maximum value of ${\sum}_{j=1}^{36585}\mathrm{Xmj}$ was selected as the best two SNPs. If the SNP combinations had the same values, the SNP combination with the second SNP located at a different chromosome as first SNP was preferentially selected as the best two SNPs. 3) Selection of the best three SNPs: a) 90 SNP combinations of the best two SNPs and each of the rest 90 SNPs were formed; b), c), and d) steps were conducted similarly as that in step 2) to select the best three SNPs. If the SNP combinations had the same values, the SNP combination with the third SNP located at different chromosome as the first and second SNPs was preferentially selected as the best three SNPs. The best 4 to 92 SNPs were selected gradually as in step 3). Discrimination power for 1 to 92 best SNPs was calculated using the following formula: discrimination power = the number of varieties showing unique genotypes / 271. The saturation curve was plotted by discrimination power for 1 to 92 best SNPs (Fig. 3). High discrimination power referred to high saturation value and high SNP discernibility.

### Core SNPs set for variety discrimination

To develop a set of core SNPs that discriminates between varieties using the KASPar platform, two allele-specific forward primers and one common reverse primer were designed for each perfect SNP marker. The 23 to 95 commercial varieties were then used to assess the potential utility of the SNP markers through the KASPar platform; fluorescence was detected as previously described [83]. Detailed instructions are available at www.kbioscience.co.uk. The Perl script, as mentioned above, was used to select a core-SNP set from the successfully verified SNP markers. Finally, the SNP markers associated with the maximum variety discrimination (the highest saturation value) were identified as a core-SNP set. The primer sequences of the core-SNP markers are shown in Additional file 11: Table S4.

### Association analysis

FSI for each variety was calculated as the ratio of maximum height to maximum width. Ninety-two SNP loci (Additional file 9: Table S2) combined with 73 SSR loci (Additional file 6: Figure S6), all developed in this study and detected by target sequencing across 271 varieties, were used for association analysis. The methods used for SSRs target library construction and detection were the same as those used in Target SNP-seq. The software program TASSEL 5.2.25 was used for association analysis. The MLM that considered both the fruit shape populations (Q matrix) and the kinship matrix (*K* matrix), and a general linear model (GLM) using fruit shape populations (Q matrix) as a fixed factor were used for association identification of loci conferring fruit shape. Significance of marker-trait association was indicated when the *p*-value was less than 10^− 4^. Because it has been popularly proved that the MLM + Q + *K* model is more effective than other models in detecting loci [84, 85], only data from the MLM + Q + K model is presented in this study. The phenotypic variation explained by each perfect SNP was the *R*^*2*^-value obtained from the MLM. Candidate genes between the nearest up- and down-stream SNP loci to the significantly associated loci were identified from the protein annotation published using the CM334 genome [3].

## Supplementary information

**Additional file 1: Figure S1.** Examples of fruit shape classification. Fruit shapes were categorized into four types as (A) blocky-fruited: blocky shape, 5.0–12.5 cm wide at the shoulder, 7.0–18 cm long, 3–4 lobes, including Fang Jiao, Chang Fang Jiao, and Ma La Jiao, as named in China; (B) long horn-fruited: long horn shape, 3.0–8.0 cm wide at the shoulder, 10.0–35.0 cm long, without lobe, including Niu Jiao Jiao, Yang Jiao Jiao, and Luo Si Jiao, as named in China; (C) short horn-fruited: cone-shaped, medium-hot, 1.0–3.0 cm in diameter at the base, 3.5–10.0 cm in length, and with very thin pericarp, including Gan Jiao and Chao Tian Jiao, as named in China; (D) linear-fruited: cayenne type, 1.0–3.0 cm wide by 10.0–35.0 cm long, without shoulder and lobe, including Xian Jiao, Tiao Jiao and Mei Ren Jiao, as named in China.

**Additional file 2: Figure S2.** Target SNP-seq genotyping analysis results. Distribution of the average read depths (A), reads alignment rate to the pepper reference genome (B), target region alignment rate (C), and uniformity for 271 pepper varieties (D).

**Additional file 3: Figure S3.** Genetic diversity analysis for the 92 perfect SNPs across 271 pepper varieties. Minor allele frequency (MAF; A), observed heterozygosity (*H*_*o*_; B), expected heterozygosity (*H*_*e*_; C), and polymorphism information content (PIC; D).

**Additional file 4: Figure S4.** Kompetitive allele-specific PCR (KASPar) results of the 35 core SNP markers genotyped across 23 to 95 pepper varieties.

**Additional file 5: Figure S5.** Significance testing of differentiation between Pops and among Subpops. The graphs show significant population differentiation at all levels given that the observed line (black) does not fall within the distribution expected of the permutation.

**Additional file 6: Figure S6.** Chromosomal map of a subset of markers used in association analysis with fruit shape index (FSI). The physical position of each marker on 12 chromosomes of *C. annuum* Zunla-1 [3] are shown between brackets. Significantly associated markers are shown in red color.

**Additional file 7: Figure S7.** Manhattan plots (A) and quantile-quantile plots (B) of fruit shape index (FSI) in the 271 pepper varieties. Red dashed line indicates high probability of associated loci with FSI.

**Additional file 8: Table S1.** Classification of and information on the pepper varieties used in this study.

**Additional file 9: Table S2.** Multiplexed primers panel of the 92 perfect SNPs used for Target SNP-seq.

**Additional file 10: Table S3.** Characteristics of the 92 perfect SNPs and the diversity detected in the 271 pepper varieties and four fruit shape populations.

**Additional file 11: Table S4.** Primer sequences of the 35 core-SNP markers developed in this study.

**Additional file 12: Table S5.** Range, mean, and standard deviations (SD) collected for fruit shape index (FSI) in the pepper varieties.

**Additional file 13: Table S6.** Association regions with fruit shape index (FSI) in the reference genomes of CM334 and Zunla-1.

**Additional file 14: Table S7.** Specific primers used to detect functional resistance loci against four pepper diseases through Target SNP-seq.

## Data Availability

The raw sequence data reported in this paper have been deposited in the Genome Sequence Archive in BIG Data Center (BIG data center members, 2019), Beijing Institute of Genomics (BIG), Chinese Academy of Sciences, under accession number CRA001576. The data are publicly accessible at http://bigd.big.ac.cn/gsa.

## Abbreviations

AFLP: Amplified fragment length polymorphism
AMOVA: Analysis of molecular variance
FSI: Fruit shape index
GATK: Genome Analysis Toolkit
GBS: Genotyping-by-sequencing
GBTS: Genotyping by target sequencing
GLM: General linear model
*He*: Expected heterozygosity
*Ho*: Observed heterozygosity
InDel: Insertion or deletion
KASPar: Kompetitive allele-specific PCR
MAF: Minor allele frequency
MLM: Mixed linear model
PCA: Principal component analysis
PIC: Polymorphism information content
RAPD: Random amplified polymorphic
RFLP: Restriction fragment length polymorphism
SNP: Single-nucleotide polymorphism
SSR: Simple sequence repeats
