# Supplementary figures and images for: Target sequencing reveals genetic diversity, population structure, core-SNP markers, and fruit shape-associated loci in pepper varieties
Source: BMC Plant Biol. 2019 Dec 23;19:578. doi: 10.1186/s12870-019-2122-2 (PMC6929450; doi:10.1186/s12870-019-2122-2)

**A**

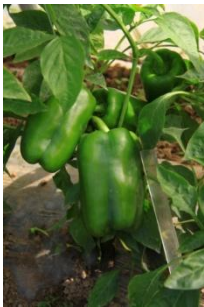

LJ-358

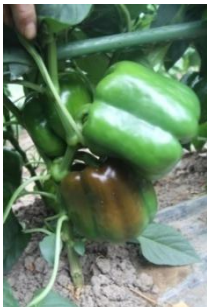

LJCX-5

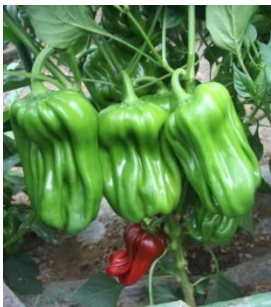

LJCX-9

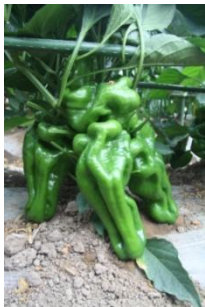

LJCX-8

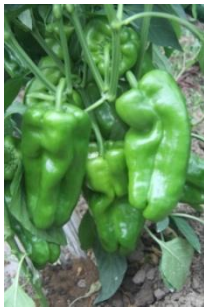

LJCX-7

**B**

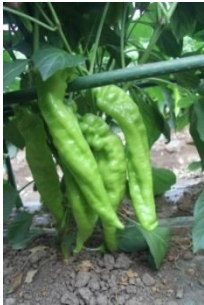

LJCX-15

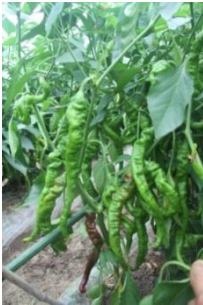

LJCX-17

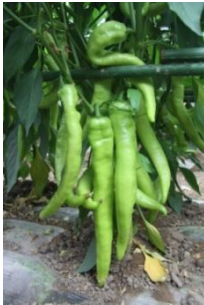

LJCX-21

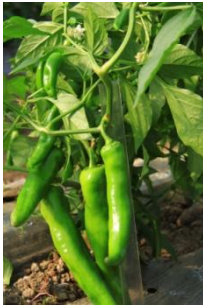

LJ-377

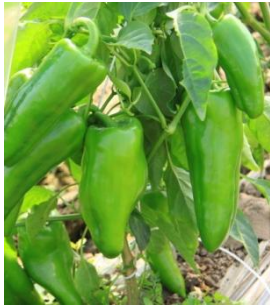

LJ-303

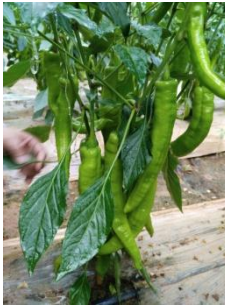

LJ-324

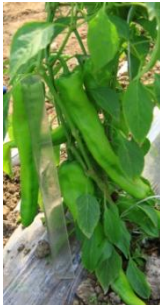

LJ-280

**C**

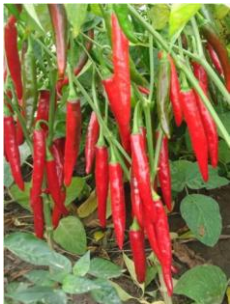

LJ-302

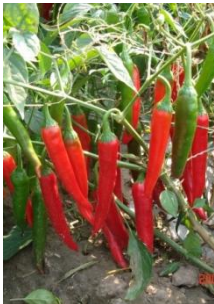

LJ-308

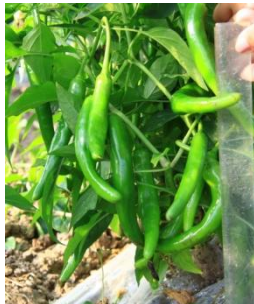

LJ-375

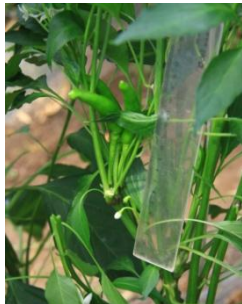

LJ-704

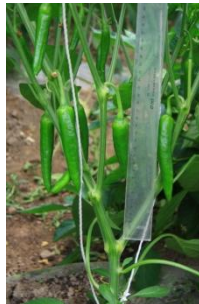

LJ-647

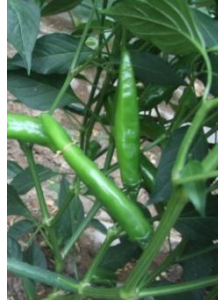

LJCX-30

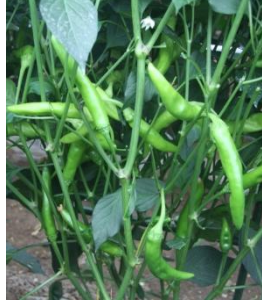

LJCX-31

**D**

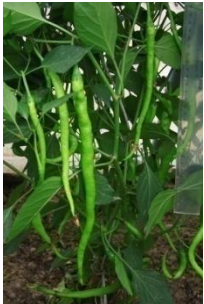

LJ-690

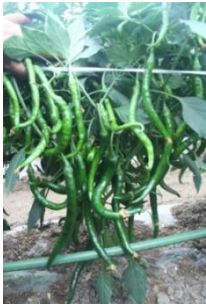

LJCX-28

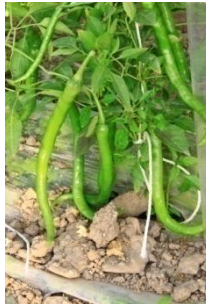

LJ-511

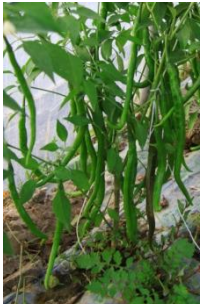

LJ-601

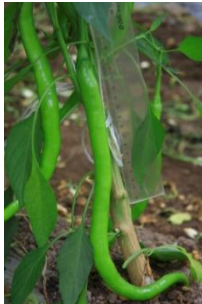

LJ-650

Supplement: Supplementary file 1 — Additional file 1: Figure S1. Examples of fruit shape classification. Fruit shapes were categorized into four types as (A) blocky-fruited: blocky shape, 5.0–12.5 cm wide at the shoulder, 7.0–18 cm long, 3–4 lobes, including Fang Jiao, Chang Fang Jiao, and Ma La Jiao, as named in China; (B) long horn-fruited: long horn shape, 3.0–8.0 cm wide at the shoulder, 10.0–35.0 cm long, without lobe, including Niu Jiao Jiao, Yang Jiao Jiao, and Luo Si Jiao, as named in China; (C) short horn-fruited: cone-shaped, medium-hot, 1.0–3.0 cm in diameter at the base, 3.5–10.0 cm in length, and with very thin pericarp, including Gan Jiao and Chao Tian Jiao, as named in China; (D) linear-fruited: cayenne type, 1.0–3.0 cm wide by 10.0–35.0 cm long, without shoulder and lobe, including Xian Jiao, Tiao Jiao and Mei Ren Jiao, as named in China. [file 12870_2019_2122_MOESM1_ESM.pdf]

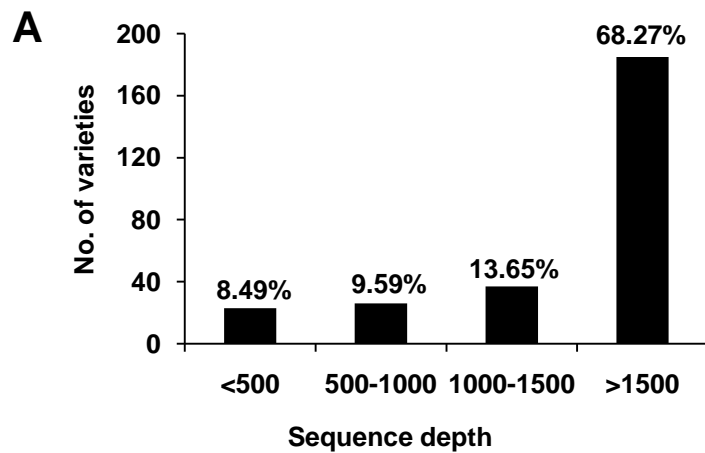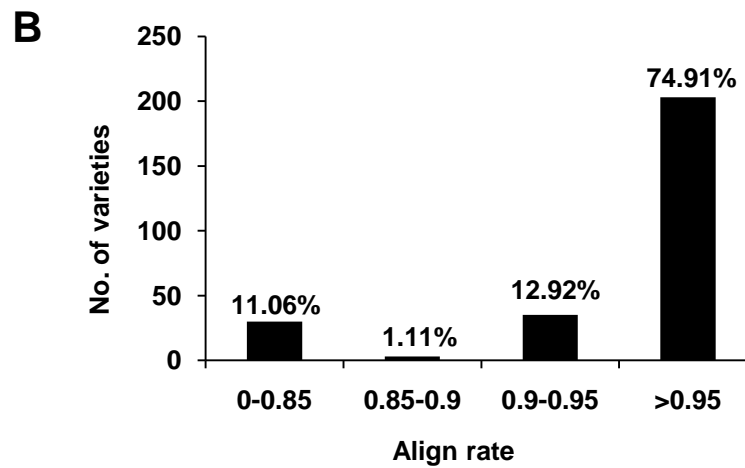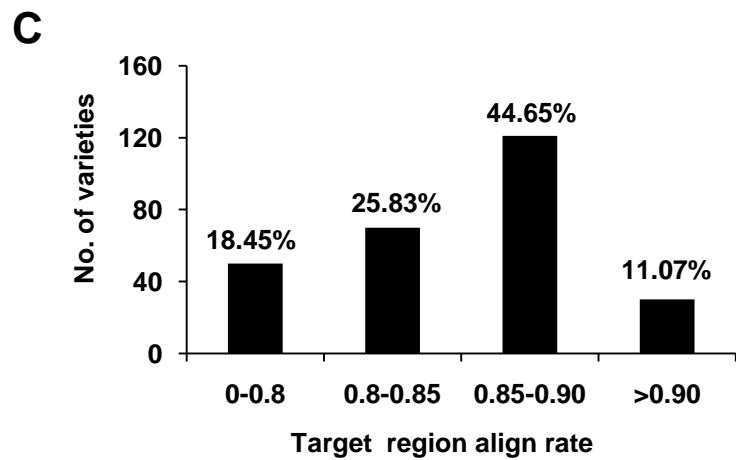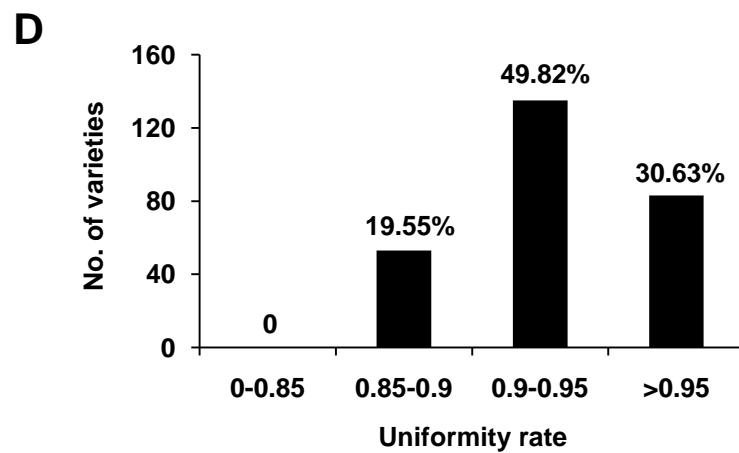

Supplement: Supplementary file 2 — Additional file 2: Figure S2. Target SNP-seq genotyping analysis results. Distribution of the average read depths (A), reads alignment rate to the pepper reference genome (B), target region alignment rate (C), and uniformity for 271 pepper varieties (D). [file 12870_2019_2122_MOESM2_ESM.pdf]

**A**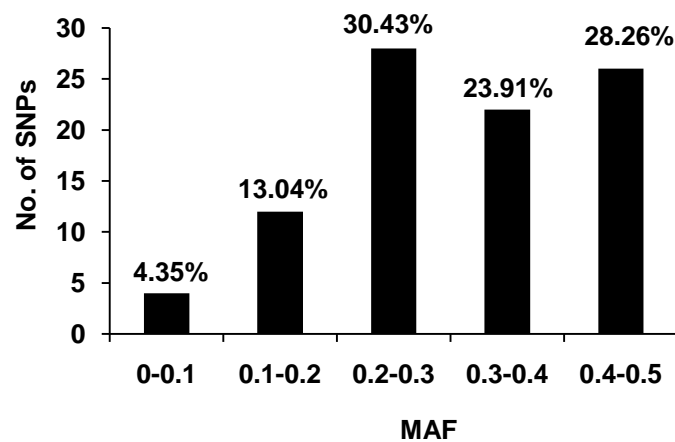**B**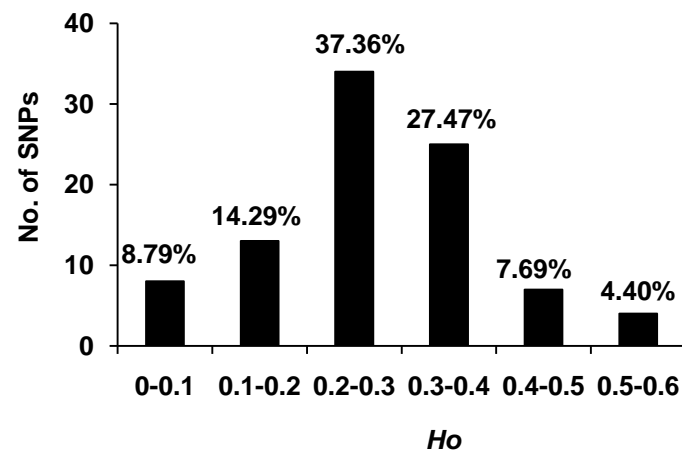**C**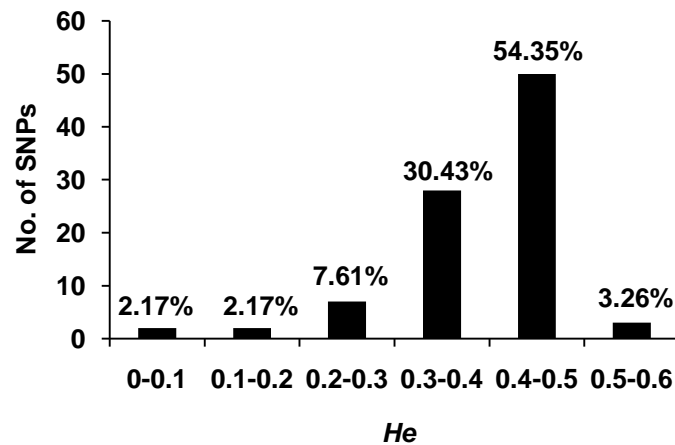**D**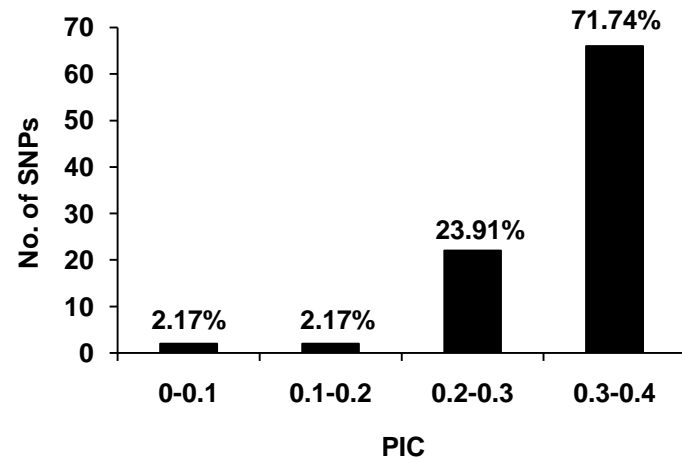

Supplement: Supplementary file 3 — Additional file 3: Figure S3. Genetic diversity analysis for the 92 perfect SNPs across 271 pepper varieties. Minor allele frequency (MAF; A), observed heterozygosity (Ho; B), expected heterozygosity (He; C), and polymorphism information content (PIC; D). [file 12870_2019_2122_MOESM3_ESM.pdf]

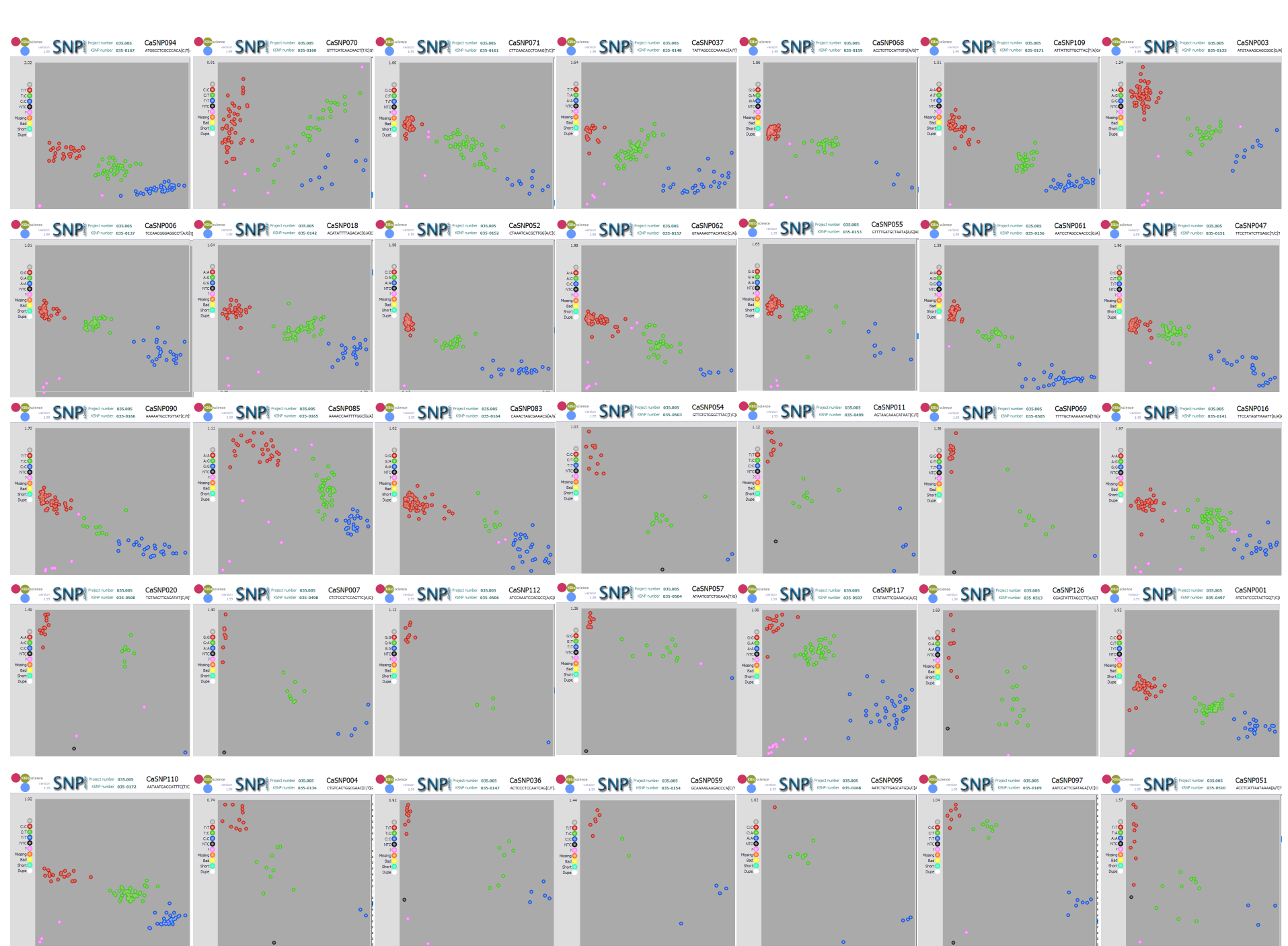

Supplement: Supplementary file 4 — Additional file 4: Figure S4. Kompetitive allele-specific PCR (KASPar) results of the 35 core SNP markers genotyped across 23 to 95 pepper varieties. [file 12870_2019_2122_MOESM4_ESM.pdf]

**A**

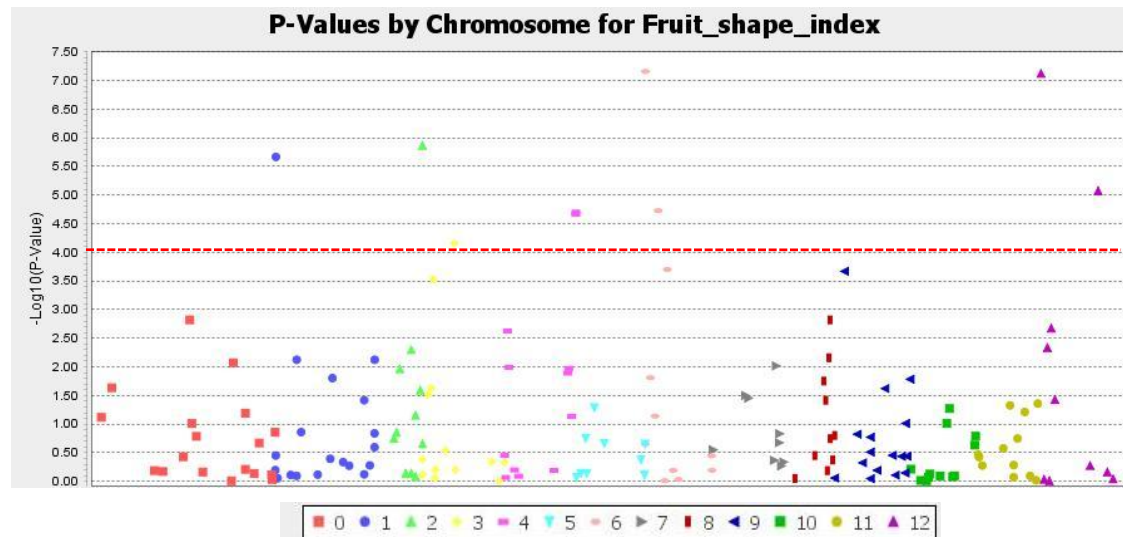

**B**

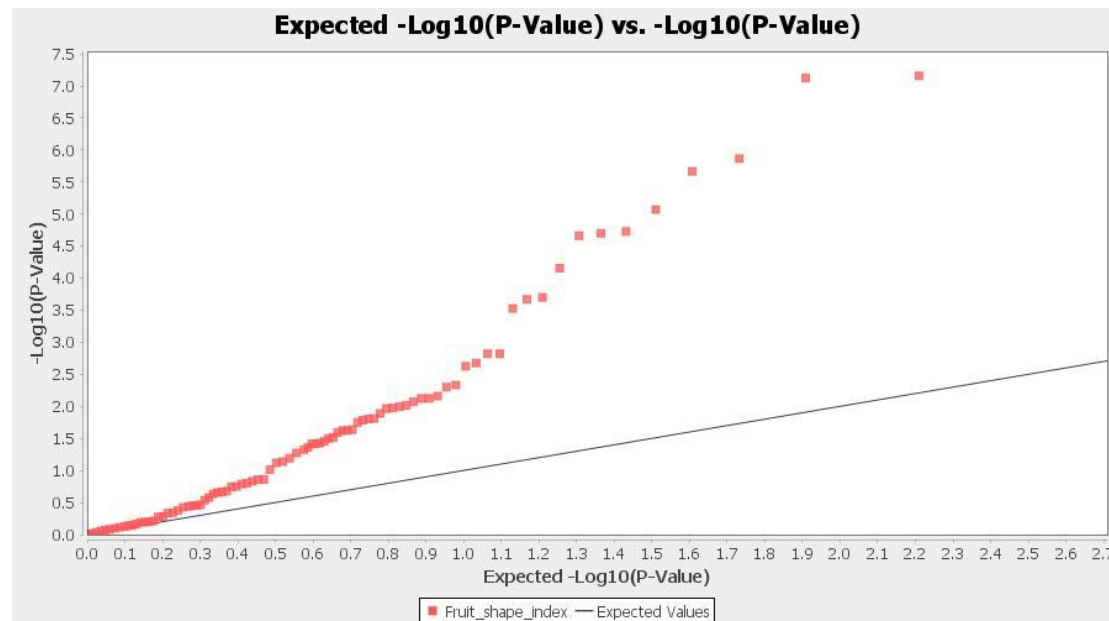

Supplement: Supplementary file 7 — Additional file 7: Figure S7. Manhattan plots (A) and quantile-quantile plots (B) of fruit shape index (FSI) in the 271 pepper varieties. Red dashed line indicates high probability of associated loci with FSI. [file 12870_2019_2122_MOESM7_ESM.pdf]
