# Supplementary material for: Target sequencing reveals genetic diversity, population structure, core-SNP markers, and fruit shape-associated loci in pepper varieties
Source: BMC Plant Biol. 2019 Dec 23;19:578. doi: 10.1186/s12870-019-2122-2 (PMC6929450; doi:10.1186/s12870-019-2122-2)
Supplement: Supplementary file 5 — Additional file 5: Figure S5. Significance testing of differentiation between Pops and among Subpops. The graphs show significant population differentiation at all levels given that the observed line (black) does not fall within the distribution expected of the permutation. [file 12870_2019_2122_MOESM5_ESM.pdf]

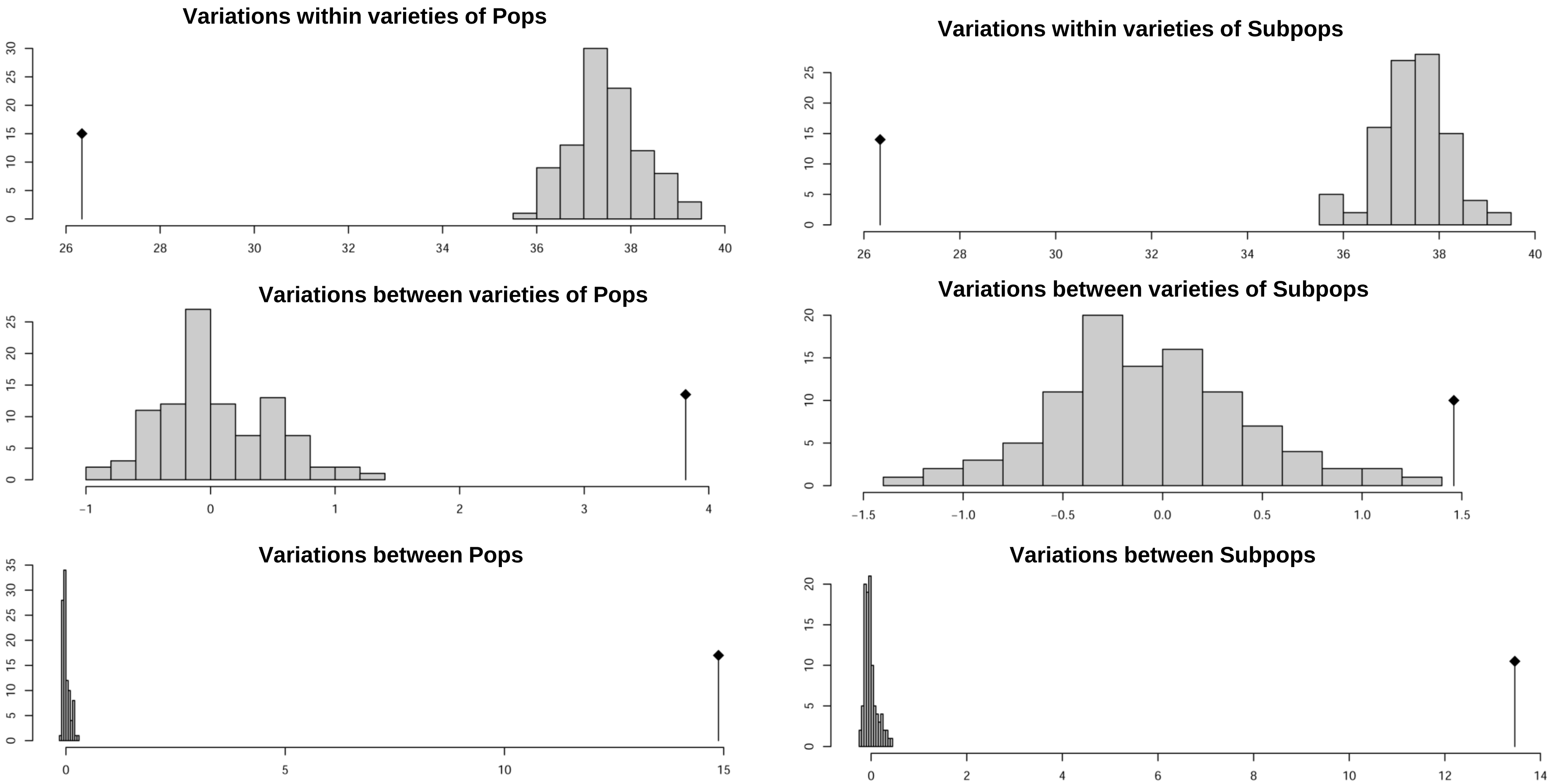
Variations within varieties of Pops
0 5 10 15 20 25 30
26 28 30 32 34 36 38 40
Variations within varieties of Subpops
0 5 10 15 20 25
26 28 30 32 34 36 38 40
Variations between varieties of Pops
0 5 10 15 20 25
−1 0 1 2 3 4
Variations between varieties of Subpops
0 5 10 15 20
−1.5 −1.0 −0.5 0.0 0.5 1.0 1.5
Variations between Pops
0 5 10 15 20 25 30 35
0 5 10 15
Variations between Subpops
0 5 10 15 20
0 2 4 6 8 10 12 14
