# Supplementary material for: Target sequencing reveals genetic diversity, population structure, core-SNP markers, and fruit shape-associated loci in pepper varieties
Source: BMC Plant Biol. 2019 Dec 23;19:578. doi: 10.1186/s12870-019-2122-2 (PMC6929450; doi:10.1186/s12870-019-2122-2)
Supplement: Supplementary file 6 — Additional file 6: Figure S6. Chromosomal map of a subset of markers used in association analysis with fruit shape index (FSI). The physical position of each marker on 12 chromosomes of C. annuum Zunla-1 [3] are shown between brackets. Significantly associated markers are shown in red color. [file 12870_2019_2122_MOESM6_ESM.pdf]

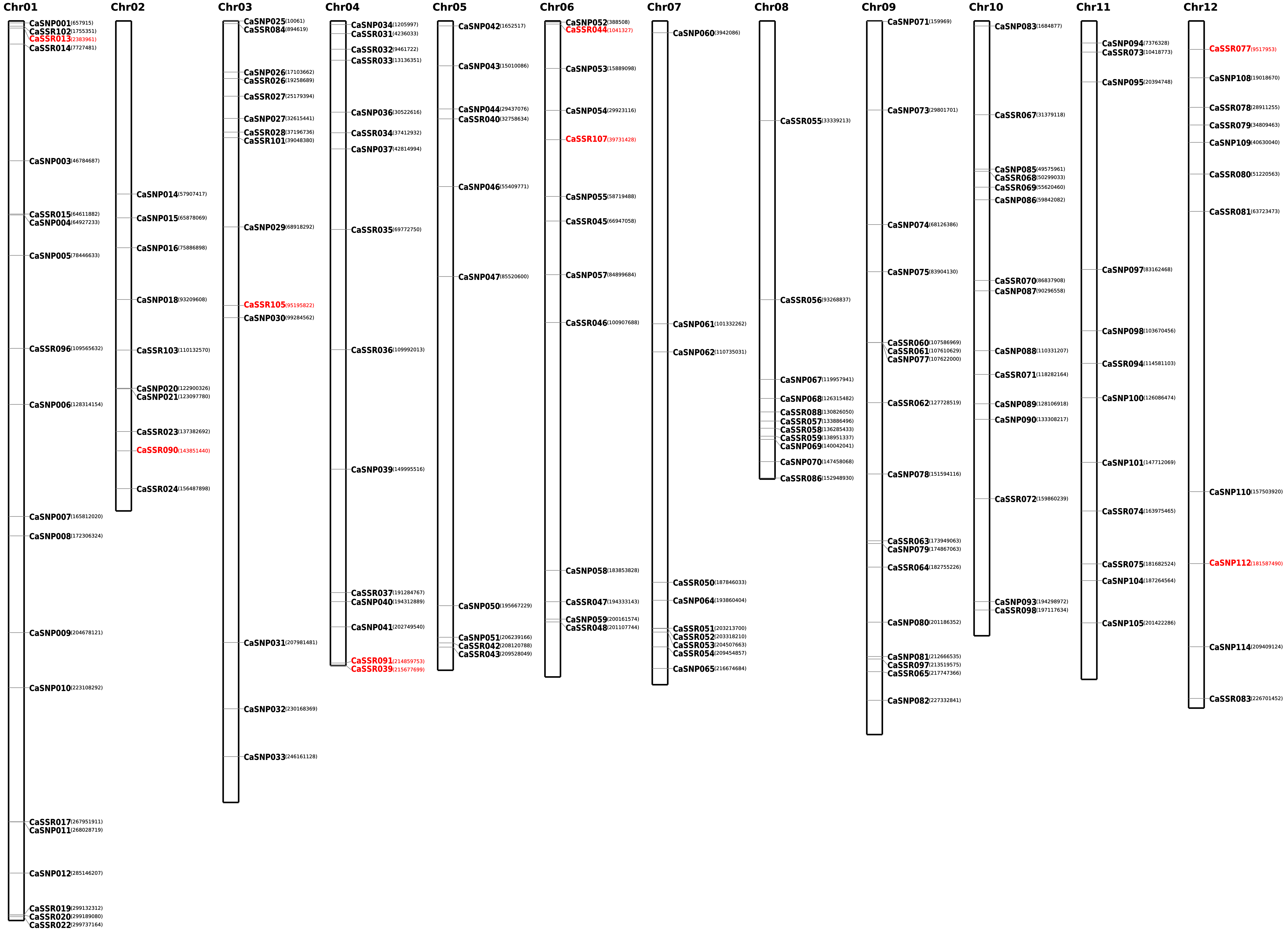

Chr01
CaSNP001(657915)
CaSSR102(1755351)
CaSSR013(2383961)
CaSSR014(7727481)
CaSNP003(46784687)
CaSSR015(64611882)
CaSNP004(64927233)
CaSNP005(78446633)
CaSSR096(109565632)
CaSNP006(128314154)
CaSNP007(165812020)
CaSNP008(172306324)
CaSNP009(204678121)
CaSNP010(223108292)
CaSSR017(267951911)
CaSNP011(268028719)
CaSNP012(285146207)
CaSNP019(299132312)
CaSSR020(299189080)
CaSSR022(299737164)
Chr02
CaSNP014(57907417)
CaSNP015(65878069)
CaSNP016(75886898)
CaSNP018(93209608)
CaSSR103(110132570)
CaSNP020(122900326)
CaSNP021(123097780)
CaSSR023(137382692)
CaSSR090(143851440)
CaSSR024(156487898)
Chr03
CaSNP025(10061)
CaSSR084(894619)
CaSNP026(17103662)
CaSSR026(19258689)
CaSSR027(25179394)
CaSNP027(32615441)
CaSSR028(37196736)
CaSSR101(39048380)
CaSNP029(68918292)
CaSSR105(95195822)
CaSNP030(99284562)
CaSNP031(207981481)
CaSNP032(230168369)
CaSNP033(246161128)
Chr04
CaSNP034(1205997)
CaSSR031(4236033)
CaSSR032(9461722)
CaSSR033(13136351)
CaSNP036(30522616)
CaSSR034(37412932)
CaSNP037(42814994)
CaSSR035(69772750)
CaSSR036(109992013)
CaSNP039(149995516)
CaSSR037(191284767)
CaSNP040(194312889)
CaSNP041(202749540)
CaSSR091(214859753)
CaSSR039(215677699)
Chr05
CaSNP042(1652517)
CaSNP043(15010086)
CaSNP044(29437076)
CaSSR040(32758634)
CaSNP046(55409771)
CaSNP047(85520600)
CaSNP050(195667229)
CaSNP051(206239166)
CaSSR042(208120788)
CaSSR043(209528049)
Chr06
CaSNP052(388508)
CaSSR044(1041327)
CaSNP053(15889098)
CaSNP054(29923116)
CaSSR107(39731428)
CaSSR055(58719488)
CaSSR045(66947058)
CaSNP057(84899684)
CaSSR046(100907688)
CaSNP058(183853828)
CaSSR047(194333143)
CaSNP059(200161574)
CaSSR048(201107744)
Chr07
CaSNP060(3942086)
CaSNP061(101332262)
CaSNP062(110735031)
CaSSR050(187846033)
CaSNP064(193860404)
CaSSR051(203213700)
CaSSR052(203318210)
CaSSR053(204507663)
CaSSR054(209454857)
CaSNP065(216674684)
Chr08
CaSSR055(33339213)
CaSSR056(93268837)
CaSNP067(119957941)
CaSNP068(126315482)
CaSSR088(130826050)
CaSSR057(133886496)
CaSSR058(136285433)
CaSSR059(138951337)
CaSNP069(140042041)
CaSNP070(147458068)
CaSSR086(152948930)
Chr09
CaSNP071(159969)
CaSNP073(29801701)
CaSNP074(68126386)
CaSNP075(83904130)
CaSSR060(107586969)
CaSSR061(107610629)
CaSNP077(107622000)
CaSSR062(127728519)
CaSNP078(151594116)
CaSSR063(173949063)
CaSNP079(174867063)
CaSSR064(182755226)
CaSNP080(201186352)
CaSNP081(212666535)
CaSSR097(213519575)
CaSSR065(217747366)
CaSNP082(227332841)
Chr10
CaSNP083(1684877)
CaSSR067(31379118)
CaSNP085(49575961)
CaSSR068(50299033)
CaSSR069(55620460)
CaSNP086(59842082)
CaSSR070(86837908)
CaSNP087(90296558)
CaSNP088(110331207)
CaSSR071(118282164)
CaSNP089(128106918)
CaSNP090(133308217)
CaSSR072(159860239)
CaSNP093(194298972)
CaSSR098(197117634)
Chr11
CaSNP094(7376328)
CaSSR073(10418773)
CaSNP095(20394748)
CaSNP097(83162468)
CaSNP098(103670456)
CaSSR094(114581103)
CaSNP100(126086474)
CaSNP101(147712069)
CaSSR074(163975465)
CaSSR075(181682524)
CaSNP104(187264564)
CaSNP105(201422286)
Chr12
CaSSR077(9517953)
CaSNP108(19018670)
CaSSR078(28911255)
CaSSR079(34809463)
CaSNP109(40630040)
CaSSR080(51220563)
CaSSR081(63723473)
CaSNP110(157503920)
CaSNP112(181587490)
CaSNP114(209409124)
CaSSR083(226701452)
